# Cardiotoxicity of Biological Therapies in Cancer Patients: An In-depth Review

**DOI:** 10.2174/1573403X18666220531094800

**Published:** 2023-03-22

**Authors:** Luai Madanat, Ruby Gupta, Paul Weber, Navneet Kumar, Rohit Chandra, Hycienth Ahaneku, Yatharth Bansal, Joseph Anderson, Abhay Bilolikar, Ishmael Jaiyesimi

**Affiliations:** 1 Department of Internal Medicine, William Beaumont Hospital, Royal Oak, Michigan;; 2 Department of Hematology and Medical Oncology, William Beaumont Hospital, Royal Oak, Michigan;; 3 College of Osteopathic Medicine, Michigan State University, East Lansing, Michigan;; 4 Department of Cardiovascular Disease, St. Joseph Mercy Oakland Hospital, Pontiac, Michigan;; 5 Department of Internal Medicine, University of Detroit Mercy, Detroit, Michigan;; 6 Department of Cardiovascular Disease, William Beaumont Hospital, Royal Oak, Michigan

**Keywords:** Cardiotoxicity, anticancer drugs, targeted therapies, biological therapies, immune checkpoint inhibitors, cardiooncology

## Abstract

Cardiotoxicity from chemotherapy regimens has been long reported. However, the understanding of cardiac side effects of biological therapies is rapidly evolving. With cancer patients achieving higher life expectancy due to the use of personalized medicine and novel targeted anticancer agents, the occurrence of cardiotoxicity is becoming more significant. Novel biological therapies include anti-HER2 antibodies, tyrosine kinase inhibitors, bruton kinase inhibitors, anti-vascular endothelial growth factors, proteasome inhibitors, immunomodulator drugs, and immune checkpoint inhibitors. Potential cardiovascular toxicities linked to these anticancer agents include hypertension, arrhythmias, QT prolongation, myocardial ischemia and infarction, left ventricular dysfunction, congestive heart failure, and thromboembolism. Cardiac biomarkers, electrocardiography, echocardiography and magnetic resonance imaging are common diagnostic modalities used for early detection of these complications and timely intervention. This review discusses the various types of cardiotoxicities caused by novel anticancer biologic agents, their molecular and pathophysiological mechanisms, risk factors, and diagnostic and management strategies that can be used to prevent, minimize, and treat them.

## 1 INTRODUCTION

There has been remarkable progress in the development of targeted anticancer therapies over the past decade. The targeted therapies and immune checkpoint inhibitors (ICI) have become vital components in current treatment strategies for the management of malignancies. Long-term cancer survival rates have increased due to the advent of these novel agents. Unfortunately, many of these anticancer agents contribute to morbidity and mortality due to their related cardiotoxicities. Therefore, it becomes crucial to take measures to prevent or identify cardiotoxicity early on to improve the quality of life in cancer survivors [1].

Chemotherapy-related cardiotoxicity (CRC) can be classified as either acute or chronic depending on the time of onset with regards to antineoplastic agent initiation. Acute cardiotoxicity occurs during or soon after the initiation of chemotherapy and is usually transient and self-limited [2, 3]. Molecularly targeted cancer therapies as well as immune checkpoint inhibitors have been associated with hypertension (HTN), myocyte damage, myocardial ischemia (MI), left ventricular (LV) systolic and diastolic dysfunction, congestive heart failure (CHF), thrombogenesis, pericardial disease, cardiac arrhythmias, and coronary ischemia [4]. CHF caused by anticancer agents has been linked to a 3.5-fold increase in mortality risk compared to idiopathic cardiomyopathy [5]. Despite ongoing research, we do not have a clear understanding of the molecular and pathophysiologic mechanisms, risk factors, and natural history of cardiotoxicity caused by biologics in cancer treatment. There is also a lack of evidence-based guidelines for monitoring and management of these patients due to the absence of prospective studies.

In this review, we will focus on the novel anticancer biologic agents associated with cardiovascular toxicity and the molecular mechanisms and pathophysiology leading to these effects. We will also discuss recent advances in the prevention and treatment strategies that are relevant to patient care. The goal of this review is to help improve early diagnosis and detection of such adverse effects while minimizing the cardiac morbidity and mortality associated with the use of new biologic anticancer treatments.

## 2 MOLECULAR MECHANISMS OF CARDIOTOXICITY

The European Society of Cardiology (ESC) guidelines broadly divide the cardiovascular complications of cancer therapy into nine major categories pertaining to either the cardiac or vascular system [6]. These include myocardial dysfunction and congestive heart failure (CHF), coronary artery disease, arrhythmias, valvular heart disease, and pericardial diseases. Vascular complications include arterial hypertension, peripheral vascular disease, stroke, thromboembolic events, and pulmonary hypertension [6]. Biological therapies and their molecular mechanisms of cardiotoxicity are summarized in Table **1**.

Now we will discuss the molecular mechanisms of each class of biological therapies in detail. Studies have shown that the main cause of immune checkpoint inhibitor (ICI)-associated myocarditis is infiltration of the myocardium by CD4+/CD8+ T lymphocytes and a few macrophages (CD68+ cells) [7]. The CXCL9-CXCL10-CXCL11/CXCR3 axis is known to regulate migration, differentiation, and activation of immune cells, leading to upregulation of T cell activities [8]. Finally, activated T cells cause cell death by producing granzyme B, tumor necrosis factor-α, and interferon-γ. The overexpression of these inflammatory molecules might eventually contribute to cardiotoxicity [7, 8].

The molecular mechanisms of HER2/ErbB2 signaling and their pathophysiologic mechanisms have been well studied. Cardiomyocytes are known to express ErbB2/ErbB4 receptors, which contribute to repair, growth and survival of cardiomyocytes [4]. Neuregulin-1 (NRG-1), which promotes cardiomyocyte survival *via* ErbB2/ErbB4 heterodimerization, is blocked by agents, such as trastuzumab [4]. An important mechanism of trastuzumab-induced cardiotoxicity is via disruption of the Notch and nuclear factor-κB signaling pathways that regulate cardiovascular homeostasis and play a role in regulating cardiac hypertrophy, cardiomyopathy, and heart failure [9]. Anti-HER2 therapy may also cause dysfunction of calcium homeostasis within cardiomyocytes, leading to oxidative damage within the myocardium similar to that caused by anthracyclines or other antineoplastic agents [4].

Tyrosine kinase inhibitors inhibit several different tyrosine kinases; one specific subtype is the VEGF receptor [4]. The arterial vascular tone is directly affected by these TKIs, resulting in hypertension. The underlying mechanism by which hypertension is known to occur is through nitric oxide metabolism dysfunction, endothelial damage, and vascular rarefaction [10]. Direct cardiomyocyte mitochondrial damage and cytochrome-C–induced apoptosis may cause CHF [11].

VEGF plays a significant role in vascular homeostasis as it is known to stimulate angiogenesis, cause the proliferation of endothelial cells, and prevent endothelial damage [12]. Inhibition of endothelial nitric oxide synthase is the main mechanism of action for VEGF inhibitors. This leads to reduced endothelial nitric oxide, which in turn leads to hypertension due to vasoconstriction and a decrease in sodium excretion. VEGF inhibitors cause CHF through three possible mechanisms, including a decrease in myocardial capillary density, global contractile dysfunction, and hypertension [12]. Thromboembolic events are also known side-effects of this pharmaceutical class as they cause damage to vascular endothelium, exposing the underlying collagen and triggering stimulation of tissue factors [12].

Bruton tyrosine kinase (BTK) inhibitors are known to inhibit B-cell receptor signaling that eventually stops cell proliferation and survival, playing an important role in B-cell malignancies by covalently binding to the cysteine-481 residue within the adenosine-triphosphate-binding site of BTK (PI3K-Akt pathway) [13]. In animal studies, increased PI3K activity leads to less atrial fibrosis and better cardiac conduction. Patients with atrial fibrillation have significantly lower cardiac PI3K-AKT activity, and the use of BTK attenuates the Akt response and thus predisposes to cardiac fibrosis and atrial arrhythmias, such as atrial fibrillation [13].

The ubiquitin-proteosome system is responsible for the maintenance of cellular protein homeostasis [14]. Proteasome inhibition caused by proteasome inhibitors (PI) leads to an intracellular accumulation of aggregated proteins, which in turn decreases endothelial progenitor cell proliferation, endothelial nitric oxide synthase, and increases cell apoptosis by altering transcriptional activation of NF-κB targets, thus leading to coronary vasospasm, ischemia and heart failure [15].

The exact mechanisms underlying immunomodulator-induced vascular and cardiotoxicity are not fully understood. It is postulated that alteration of equilibrium between procoagulant and anticoagulant proteins on the surface of endothelial cells may be the leading cause of thromboembolism [6]. Immunomodulators are also known to inhibit TNFα production and enhance the activity of T cells and natural killer cells, as well as enhance antibody-dependent cellular cytotoxicity [16].

## 3 CARDIOTOXICITIES OF TARGETED AGENTS

### 3.1 Immune-Checkpoint Inhibitors (ICIs)

Immune checkpoint inhibitors are a group of anticancer drugs that facilitate T-cell activation and enhance the antitumor immune response by interfering with immune checkpoint molecules, primarily cytotoxic T-lymphocyte antigen 4 (CTLA-4), programmed cell death receptor-1 (PD-1) and programmed cell death ligand-1 (PD-L1) [17, 18]. Monoclonal antibodies targeting these checkpoint proteins include the PD-1 inhibitors nivolumab and pembrolizumab, PD-L1 inhibitors atezolizumab, avelumab, and durvalumab, and CTLA-4 inhibitors ipilimumab and tremelimumab.

The development of ICIs has marked a new era in the field of oncology, with studies reporting unprecedented success in the treatment of a broad spectrum of solid and hematological tumors [19-21]. However, their increased use in the setting of malignancy has led to a corresponding increase in side effects, better known as immune-related adverse events (IRAEs) [22-24].

ICI-associated cardiotoxicity has gained interest since it was first described in 2014, with more recent studies reporting an increasing number of patients affected by this phenomenon [25-31]. Cardiac effects associated with ICIs include a wide array of pathologies, including myocarditis, pericarditis, LV dysfunction, takotsubo-like syndrome, arrhythmias, and ischemia [32-35]. Myocarditis is the most common pathology with a prevalence of 0.06%-2.4%, and with combination therapy posing the greatest risk [35-37]. Several retrospective studies have demonstrated the occurrence of fulminant myocarditis with ICI use leading to fatality despite intensive medical therapy [38, 39].

The global database (Vigibase) of the World Health Organization (WHO) was analyzed by Salem *et al.* in one of the largest studies to date reporting ICI-associated cardiotoxicity. Myocarditis occurred at an 11 times greater rate in patients treated with ICIs compared to those without ICI treatment [34]. The study also reported significantly higher fatality rates up to 46% in patients treated with combination therapy. A similar study published in 2019 also assessed the frequency of IRAEs related to ICIs through an analysis of Vigibase. Cardiotoxicity regardless of type was reported in 1.99% of patients treated with pembrolizumab, 2.23% with nivolumab, 2.59% with atezolizumab, 3.17% with avelumab, 2.56% with durvalumab, and 1.81% with ipilimumab [39].

A meta-analysis published by Wang *et al.* in 2018 evaluated IRAEs related to ICIs by analyzing data from the WHO pharmacovigilance database (Vigilyze) [40]. 613 ICI-related fatal events were identified from the time period from 2009 through January 2018, of which 52 were cardiac IRAEs. The majority were caused by anti-PD-1/PD-L1 antibody treatment, followed by a combination of anti-CTLA-4 and anti-PD-1/PD-L1 antibody and CTLA-4 antibody. Of all fatal side effects reported, ICI-related myocarditis had the highest mortality rate at 39.7% [40].

### 3.2 HER-2 Targeted Therapies

Human epidermal growth factor receptor 2 (HER2) is a transmembrane tyrosine kinase receptor encoded by ErbB2 proto-oncogene. Over-expression of the HER2 gene is observed in 25-30% of all breast cancers and is associated with an aggressive clinical course, higher rates of disease recurrence, and increased overall mortality. Therefore, HER2-targeted therapies play an important role in breast cancer therapy, which include trastuzumab, pertuzumab, lapatinib, and ado-trastuzumab emtansine [41, 42].

#### 3.2.1 Trastuzumab

Trastuzumab is a humanized monoclonal antibody that targets HER2 by binding to its extracellular domain. The degree of cardiac dysfunction associated with trastuzumab is highly variable and ranges from asymptomatic LV dysfunction to overt CHF, with incidences more likely to occur after 6 months of continued administration [43]. Unlike anthracycline-induced CRC, trastuzumab-related cardiac dysfunction does not seem to be related to cumulative dose and is often reversible, with the resolution of symptoms reported to occur within 6 weeks of discontinuation [43, 44]. Newer studies suggest persistent symptoms in patients with trastuzumab-induced cardiac injury years after termination of the therapy [45].

Several trials have denoted increased risk of severe heart failure defined as New York Heart Association (NYHA) class III or IV in patients receiving trastuzumab compared to placebo, with the NSABP trial reporting incidents occurring in 4.1% and 0.8% of patients, respectively [46, 47]. Trastuzumab significantly increased the risk of cardiac injury when combined with other chemotherapeutic agents, with concurrent anthracyclines posing the greatest risk [48]. Studies have also demonstrated an increased risk of cardiotoxicity with anthracycline and trastuzumab combined (27%) compared to paclitaxel and trastuzumab (13%) or trastuzumab alone (7%) [49].

Other cardiac complications related to trastuzumab therapy outside of LV dysfunction include new T-wave inversions and right or left bundle branch block on electrocardiogram (ECG) [50]. Risk factors associated with increased trastuzumab-induced CRC included individuals with coexisting cardiovascular disease, diabetes mellitus or renal impairment, antihypertensive therapy, and prior history of mediastinal irradiation [51, 52].

#### 3.2.2 Pertuzumab

Pertuzumab is a recombinant monoclonal antibody that binds to a different domain than trastuzumab and works by preventing HER2 homodimerization and heterodimerization with other HER family receptors [4]. Previous studies have demonstrated no major risk of cardiotoxicity when pertuzumab is used alone or in combination with other anti-HER2 agents [53, 54]. In a recent meta-analysis, pertuzumab was shown to increase the risk of heart failure by two folds, but the study concludes that its use is safe in patients with reduced left ventricular systolic function and low cardiac risk [55].

#### 3.2.3 Lapatinib

Lapatinib is an oral reversible dual tyrosine kinase inhibitor that targets both the epidermal growth factor receptor (EGFR) and HER2, used in the treatment of HER2 positive metastatic breast cancer [56]. The cardiotoxic side effects of lapatinib were studied in a large retrospective review published by Perez *et al.* involving 3,689 patients enrolled in 44 clinical trials [57]. The incidence of cardiotoxicity was 1.6%, which included an asymptomatic decline in LVEF by more than 20% relative to patient’s baseline or symptomatic CHF. Only 0.2% of patients who experienced a cardiac event developed symptomatic CHF. Cardiac events were mostly reversible, with 88% having partial or full recovery within 7 weeks, regardless of whether or not lapatinib was continued [57]. Data also demonstrate that combination therapy using trastuzumab and lapatinib does not appear to be associated with increased cardiotoxicity compared to trastuzumab alone [58].

#### 3.2.4 Ado-trastuzumab Emtansine

Ado-trastuzumab emtansine (T-DM1) is an antibody-drug conjugate that targets HER2-positive tumors and is mainly used in patients with metastatic disease who progressed on trastuzumab [58, 59]. In the EMELIA study, a phase III trial of trastuzumab emtansine versus capecitabine and lapatinib in participants with HER2‐positive locally advanced or metastatic breast cancer, 1.7% of the 481 patients in the ado-trastuzumab group developed an LVEF<50% and at least 15% below baseline versus 1.6% in the lapatinib and capecitabine group [60].

### 3.3 Tyrosine Kinase Inhibitors

Tyrosine kinases are important enzymes in cell growth, differentiation, and apoptosis that are responsible for phosphorylating substrates to maintain homeostasis. TKIs, including imatinib, dasatinib, nilotinib, and ponatnib, have been shown to induce cardiac dysfunction during and following therapy.

#### 3.3.1 Imatinib

Imatinib is a small tyrosine kinase inhibitor that inhibits multiple tyrosine kinase receptors (ABL, BCR-ABL, C-kit) that are active in malignancy [61]. Imatinib induction has been shown to cause new-onset congestive systolic heart failure, as noted by Atallah *et al*. [62]. CHF did not correlate with imatinib dosing and occurred in 1.8% of patients in a 1,276 patient trial. The decrease in ejection fraction associated with imatinib induction was as large as 20%. In the same study, 0.6% of patients reported having a cardiac event [62]. Pericardial effusion was also described with imatinib use and was reported to occur in up to 6% of patients [63]. Imatinib is contraindicated in cases of hypereosinophilic syndrome with cardiac involvement, as it can worsen cardiac function and induce cardiac shock by causing degranulation [63].

#### 3.3.2 Dasatanib

Dasatinib is an oral TKI created in 2006 as a medication for those unable to tolerate imatinib or having imatinib-resistant Ph+ CML [64]. After dasatinib’s approval to treat CML by the FDA, Brave *et al*. completed a four-arm multicenter trial with 911 patients, which showed fluid retention in 50% of patients. Of those patients, 4% of reported new systolic heart failure and 4% of patients had new pericardial effusions. Desatinib was also associated with the development of cardiac arrhythmias in up to 11% of patients, including non-dose dependent QTc prolongation [65].

#### 3.3.3 Nilotinib

Nilotinib is an orally bioavailable TKI created shortly after imatinib to treat Ph+ CML [66]. Peripheral occlusive arterial disease is reported to occur in 1.4% of patients treated with nolitinib [67]. Ischemic cardiac disease and QTc prolongation are reported to occur in up to 7.2% and 2.5% of patients receiving nilotinib, respectively [67].

### 3.4 Vascular Endothelial Growth Factor (VEGF) Inhibitors

VEGF is a signaling protein responsible for initiating angiogenesis and is expressed in excess as tumors migrate and develop vasculature [68]. Therapies targeting VEGF include bevacizumab, sorafenib, and sunitinib [68, 69].

#### 3.4.1 Bevacizumab

Bevacizumab is a humanized anti-VEGF monoclonal IgG1 antibody that binds circulating VEGF, preventing angiogenesis and tumor growth [69]. Several trials have shown new-onset hypertension with bevacizumab, with one phase II trial showing an incidence of hypertension in 22% of patients [70]. Malignant hypertension (>200/110 mmHg) was also described in 7% of patients treated with bevacizumab [70, 71]. Data suggest that the risk of hypertension correlates with the dose exposure of bevacizumab [71]. A proposed mechanism for bevacizumab-induced hypertension is a reduction in the production of nitric oxide through VEGF inhibition, leading to increased vascular resistance [71]. The presence of hypertension is a well-established adverse effect of bevacizumab. Over time, increased afterload leads to left ventricular dysfunction and negative cardiac remodeling precipitating congestive heart failure in these patients. It is reported that 2-4% of patients receiving bevacizumab will develop congestive heart failure [71]. Patients treated with bevacizumab have been shown to have an increased risk for acute thromboembolic events (ATE), such as myocardial or cerebral ischemia/infarct and arterial thrombosis or angina [72]. In a large multicenter trial involving 1,745 patients, those who received bevacizumab demonstrated a 3.8% risk of developing an ATE following treatment [72]. Other studies have substantiated this increased risk of ATE with bevacizumab, demonstrating a relative risk of 1.44 to develop ATE compared to those not receiving treatment [73].

#### 3.4.2 Sorafenib

Sorafenib is an oral kinase inhibitor of VEGF-R2/3, with activity against PDGF-R, RAS/RAF, and c-Kit. The targeted therapy reduces the progression of malignancy by inhibiting angiogenesis [74]. Similar to bevacizumab, sorafenib has been primarily associated with hypertension in 17% of patients receiving treatment [75]. Hypertension usually manifests within the first few cycles of treatment [75, 76]. Sorafenib has also been associated with cardiac ischemia and infarction in about 3% of patients undergoing treatment [75].

#### 3.4.3 Sunitinib

Sunitinib is a tyrosine kinase inhibitor that targets multiple VEGFR receptors, platelet-derived growth factor receptor (PDGFR), and CKIT. Inhibiting these signal transduction pathways leads to decreased angiogenesis and slowed growth of malignancy [77]. There have been reports of hypertension associated with sunitinib treatment, similar to sorafenib and bevacizumab. Hypertension has been estimated to occur in 15% to 47% of patients receiving treatment with sunitinib [78]. Significant reductions in LVEF after sunitinib treatment were reported by Chu et al., with 28% of treated patients having >10% decrease in LVEF [78]. New symptomatic congestive heart failure has been reported in 8%-15% of patients after the initiation of sunitinib treatment [79]. In one report, sunitinib was associated with dilated cardiomyopathy within 3 months of initiation. LVEF recovered following discontinuation [80].

### 3.5 Bruton Kinase Inhibitors

The development of Bruton Tyrosine Kinase (BTK) Inhibitors (BKIs) has led to significant advancements in the treatment of multiple B-cell malignancies. Ibrutinib, the first-in-class agent, is used in the treatment of chronic lymphocytic leukemia (CLL), mantle cell lymphoma (MCL), small lymphocytic lymphoma, and Waldenström's macroglobulinemia [81]. Acalabrutinib is an oral, selective, irreversible inhibitor of BTK approved for the treatment of CLL and MCL [82]. Zanubrutinib is a newer BKI approved for refractory MCL treatment [83].

#### 3.5.1 Ibrutinib

As ibrutinib use has increased, atrial fibrillation (AF) has emerged as an adverse event in patients receiving this treatment, with studies reporting occurrence in 6-16% of patients [84, 85]. AF was the leading cause of ibrutinib therapy discontinuation in a retrospective cohort published by Mato *et al*. [86]. A meta-analysis involving four clinical trials evaluated the risk of ibrutinib-induced atrial fibrillation [87]. Patients with CLL, MCL and small cell lymphoma were assigned to treatment with ibrutinib vs. non-ibrutinib therapy. The pooled RR (95% CI) of atrial fibrillation associated with ibrutinib as compared to non-ibrutinib therapy was 3.9 (2.0-7.5), *p<*0.0001 [87]. A study including 53 patients published in 2019 demonstrated a higher incidence of atrial fibrillation in patients treated with ibrutinib compared to previous data [88]. The cumulative incidence of ibrutinib-induced atrial fibrillation was 21%, 23%, and 38% at 6, 12 and 24 months, respectively [88]. Several other cardiotoxicities have been reported with ibrutinib use. According to the WHO global database of individual case safety reports, supraventricular arrhythmias were reported in 7% of patients receiving ibrutinib, ventricular arrhythmias in 0.5%, heart failure in 2.7% and hypertension in 2.2% [89]. Several independent risk factors have been linked to the development of de novo AF in patients with CLL treated with ibrutinib, including older age, male gender, pre-existing valvular disease and hypertension [90].

#### 3.5.2 Zanubrutinib

Zanubrutinib has also been linked to cardiotoxicity, with an incidence of AF and atrial flutter reported at around 2%. Patient with hypertension, acute infections or other cardiac risk factors may be at increased risk of cardiotoxicity [91].

### 3.6 Proteasome Inhibitors

Proteasome inhibitors (PIs) have become the standard of care in the management of patients with multiple myeloma. Bortezomib is used predominantly in the first-line setting, while second-generation carfilzomib has been quite effective in relapsed/refractory setting [92, 93].

#### 3.6.1 Bortezomib

There have been several case reports and series demonstrating cardiotoxicity from bortezomib leading to ischemic heart disease, heart failure, and complete heart block [94-96]. However, one of the larger studies, the APEX trial, failed to show a significant difference in cardiotoxicity between the bortezomib and non-bortezomib groups [97]. A meta-analysis published in 2014 evaluating bortezomib use in cancer treatment also failed to show significantly increased cardiotoxic effects [98].

#### 3.6.2 Carfilzomib

The ASPIRE trial compared the use of carfilzomib in combination with lenalidomide and dexamethasone (KRd) to lenalidomide and dexamethasone alone (Rd); KRd group had a higher number of patients with cardiotoxicity when compared to Rd [99]. The incidence of hypertension was 14.3% in the KRd group versus 6.9% in the Rd group. The rate of heart failure rate was 3.8% for KRd compared to 1.8% for Rd, and ischemic heart disease rates were 3.3% for KRd compared to 2.1% in the Rd group [99]. In the ENDEAVOR trial, a higher incidence of cardiotoxicity, including hypertension, cardiac failure and dyspnea, was found in the carfilzomib (kd) group compared to the bortezomib group (Vd), while ischemic heart disease incidence was similar in both the groups [100]. The incidence of deep vein thrombosis was higher in carfilzomib plus dexamethasone (Kd) recipients compared to bortezomib plus dexamethasone (Vd) recipients, 3.7% versus 0.9%, respectively. Pulmonary embolism was also higher, 2.6% in the Kd group versus 0.9% in Vd recipients [100].

### 3.7 Immunomodulatory Drugs

Immunomodulatory drugs are now standard-of-care for patients with newly diagnosed multiple myeloma (MM) and those with relapsed refractory MM [101]. The first-generation immunomodulators include lenalidomide and thalidomide and the second-generation includes pomalidomide.

#### 3.7.1 Lenalidomide and Thalidomide

There have been rare reports of serious cardiac damage with these agents, although both have been associated with cardiac arrhythmias, specifically sinus bradycardia [102]. Thalidomide has also been associated with an increased risk of thromboembolism, especially when combined with dexamethasone. In a pooled analysis of two randomized trials comparing thalidomide and dexamethasone with placebo and dexamethasone, the incidence of VTE was five times higher in the thalidomide arm [103]. In one meta-analysis of patients with multiple myeloma, patients with newly diagnosed or previously treated MM thalidomide- or lenalidomide-based regimens in combination with dexamethasone were associated with a high risk of VTE [104].

#### 3.7.2 Pomalidomide

In a study comparing pomalidomide plus low-dose dexamethasone to high-dose dexamethasone alone, pomalidomide was associated with an increased risk for VTE [105]. Among the patients who received thromboprophylaxis, patients in the pomalidomide plus low-dose dexamethasone group had a higher incidence of VTE compared to the high-dose dexamethasone group [105].

Proposed adequate thromboprophylaxis to prevent thalidomide- and lenalidomide-associated thrombosis in low-risk patients consists of aspirin 100 mg daily. If more than one risk factor is present, low molecular weight heparin or full-dose warfarin should be used and continued for at least 4 months; subsequent transition to aspirin may be an option [106].

## 4 CARDIAC EVALUATION AND TREATMENT

A basic cardiac evaluation with physical examination, EKG and echocardiogram remain the standard method to establish a baseline cardiac function for those undergoing potential cardiotoxic treatment. Patients are also recommended to be screened for hypertension, diabetes, and dyslipidemia to optimize these cardiac risk factors prior to initiation of cancer treatment [107].

The American Society of Clinical Oncology (ASCO) recommends monitoring LV function when receiving biologics, most commonly HER2 targeted therapy, followed by proteasome and VEGF inhibitors [108]. Patients undergoing HER2 targeted therapy should have the same goals with regards to lipid and blood pressure parameters as HF patients in an attempt to minimize the risk of LV dysfunction [108]. Data exist regarding the use of serial troponin I and myeloperoxidase (MPO) for monitoring the development of cardiotoxicity in patients receiving trastuzumab [109]. There are no formal recommendations from the American Heart Association (AHA) or the American College of Cardiology (ACC) for the diagnosis and treatment of these patients. At this time, both organizations recommend following the recommendations of ASCO.

In patients receiving bevacizumab, recommendations are to withhold initiation of therapy if blood pressure is >160/100 until medically managed with antihypertensives [110]. Once bevacizumab is initiated, blood pressure should be measured prior to every chemotherapy infusion, and if blood pressure rises above 160/100, antihypertensive therapy should be initiated for the remainder of bevacizumab treatment [110].

Currently, there are no clear guidelines for the diagnosis of ICI-related myocarditis. Symptoms are usually broad and include dyspnea, chest pain, palpitations, dizziness and fatigue. Severe fulminant cases of myocarditis can present more dramatically with complete heart block ventricular arrythmias or cardiac arrest [36, 111]. Initial evaluation should include EKG, echocardiogram and cardiac markers; however, findings are often non-specific [36]. LVEF is most often preserved in ICI-associated myocarditis, and severe systolic dysfunction (EF <35%) is less common [112, 113]. Cardiac MRI with tissue characterization and parametric mapping can help establish the diagnosis [114]. Studies have demonstrated lower late gadolinium enhancement (LGE) with ICI-associated myocarditis compared to cases of non-ICI myocarditis [115, 116]. Endomyocardial biopsy (EMB) remains the gold standard for diagnosis of myocarditis; however, given the potential complications, it is not commonly used as first-line in establishing a diagnosis. Most reports of immunohistochemical staining predominantly show a CD8+ T cell infiltration intermixed with subsets of CD4+ T cells and CD68+ monocyte/macrophage lineages [117]. Data regarding ICI-associated myocarditis in EMB are poor and further research is needed to establish the criteria for pathological-based diagnosis.

The ASCO issued a practice guideline for the management of ICI-associated IRAEs [118]. For cardiac-related IRAEs, the consensus is to permanently discontinue therapy regardless of severity, given the high complication and mortality rates. Specific treatment is recommended based on severity, which is divided into four groups. G1 are asymptomatic cases with abnormal cardiac biomarkers or EKG, and no specific treatments are warranted. For G2 and G3, patients are generally symptomatic with abnormal testing; prompt treatment with 1-2 mg/kg of prednisone is recommended with possible hospital admission and in-patient evaluation and treatment for heart failure based on ACC/AHA guidelines. G4 are severe decompensated cases that require intensive care monitoring and treatment with transplant rejection doses of corticosteroids (methylprednisolone 1 g every day), and the addition of either mycophenolate, infliximab, or antithymocyte globulin may be considered [118].

Systematic data regarding the use of corticosteroids in ICI-mediated cardiotoxicity are lacking, and thus, the dose of steroids and duration of therapy vary among the reports. Several previous investigations have shown little benefit with corticosteroids in terms of overall survival [119, 120]. Newer investigations, however, have shown survival benefits with the use of high-dose steroids [121, 122]. In severe cases where patients are unstable and little benefit is observed with steroids, other options should be considered and include abatacept, belatacept, alemtuzumab, antithymocyte globulin or intravenous immunoglobulin [123-125]. Infliximab has also been considered as a second-line treatment [37]. However, its use in patients with reduced LV function should be avoided [126].

In all patients who develop reduced LV function and congestive heart failure, guideline-directed medical treatment for heart failure should be initiated based on ACC/AHA guidelines, including loop diuretics, ACE inhibitors, or angiotensin receptor blockers and beta-blockers [118]. If ischemia is suspected as an etiology for heart failure, urgent cardiac catheterization and revascularization, if needed, are indicated [118]. Cases of severe myocarditis warrant intensive care under the supervision of a heart failure specialist in collaboration with an oncologist [117].

## CONCLUSION

The field of cardio-oncology is rapidly evolving, with biologic targeted therapy changing the face of cancer treatment. With new and emerging therapies, the risk of intricate cardiotoxicities among other multisystem interactions arises, and our duty as physicians is to better understand the risk factors the predispose patients to developing cardiac injury and provide effective preventative and treatment solutions. Future focus should be placed on conducting prospective studies comparing current treatment modalities to establish clear guidelines on the management of targeted therapy-associated cardiotoxicity. A multidisciplinary approach is crucial to provide care and limit adverse outcomes.

## Figures and Tables

**Table 1:** Summary of novel biological anti-cancer therapies and their molecular mechanisms of cardiotoxicity.

**Biological Therapy**	**Molecular Mechanisms of Cardiotoxicity**
Immune-checkpoint Inhibitors [7-10]	Infiltration of predominant CD4+/CD8+ T lymphocytes in cardiac muscle Upregulation of CXCR3–CXCL9/CXCL10 and CCR5/CCL5 Production of tumor necrosis factor-α, granzyme B, and interferon-γ by activated T cells
HER 2/ ERB2 antibody [6]	Inhibit pro-survival NRG-1/ErbB pathway Generate reactive oxygen species
TKI/VEGFR antibody [6, 11-13]	Inhibit angiogenesis Cause endothelial dysfunction Cause energy depletion
Bruton Kinase Inhibitors [14]	Alter gene expression in ventricular tissue Compromised regulation of autonomic calcium oscillations in cardiac cells to alter automaticity Compromised role in cardiac remodeling under pressure overload or stress Attenuated activation of PLCγ-2 compromises calcium signaling
Proteosome Inhibitors [15, 16]	Intracellular accumulation of aggregated proteins disproportionally toxic to cardiac myocytes Protein homeostasis is not maintained, potentially leading to heart failure Ubiquitin–proteasome system is dysfunctional during myocardial ischemia
Immunomodulatory drugs [8, 17]	Alter the equilibrium between procoagulant and anticoagulant proteins on the surface of endothelial cells Inhibition of angiogenesis

## LIST OF ABBREVIATIONS

ICI: Immune Checkpoint Inhibitors
HTN: Hypertension
MI: Myocardial Ischemia
CHF: Congestive Heart Failure
LV: Left Ventricular
CRC: Chemotherapy-Related Cardiotoxicity
